# Composite Burkitt Lymphoma and Classical Hodgkin Lymphoma in an Human Immunodeficiency Virus (HIV)-Positive Patient

**DOI:** 10.7759/cureus.62827

**Published:** 2024-06-21

**Authors:** Dwight Smith Jr., Devaun M Reid, Abraham A Mascio, Britannia O Noel, Martin Giangreco

**Affiliations:** 1 Internal Medicine, University of South Florida Morsani College of Medicine, Tampa, USA

**Keywords:** c-myc oncogene, rare cancers, hiv lymphoma, classic hodgkin lymphoma, burkitt

## Abstract

Lymphoma, a term encompassing tumor masses in the lymph nodes, is often classified into Hodgkin and non-Hodgkin lymphomas, each with distinct subtypes. We present the unique case of an HIV-positive patient diagnosed with Burkitt lymphoma and classical Hodgkin lymphoma simultaneously as a composite lymphoma. Over the course of five years, a variety of dose-adjusted chemotherapy regimens were used that ultimately proved highly effective. The successful management of this rare case reinforces the significance of considering unexpected combinations of neoplastic processes during diagnosis and treatment planning.

## Introduction

Lymphoma, a hematologic malignancy originating in the lymph nodes, exhibits diverse etiologies based on its subtype: Hodgkin or non-Hodgkin lymphoma [1]. The pathogenesis of Hodgkin lymphoma is classically associated with risk factors such as the Epstein-Barr virus (EBV) and immunodeficiency due to human immunodeficiency virus (HIV) [2]. Classical Hodgkin lymphoma is always diagnosed using at least a lymph node biopsy with immunochemistry, which reveals the presence of the pathognomonic Reed-Sternberg cells surrounded by inflammatory and immune cell infiltrate. These B-cell-derived malignancies appear basophilic with bilobulated nuclei on biopsy, but immunohistochemistry analysis for markers variably expressed by these cells, including CD30, CD15, CD20, and paired box protein 5 (PAX5), is used to confirm the diagnosis [2]. The disease typically manifests with clinical symptoms such as lymphadenopathy, more in the neck, low-grade fever, and weight loss, and is most often diagnosed in individuals in their mid-thirties [2]. 

In contrast, Burkitt lymphoma (BL) is a well-recognized subtype of non-Hodgkin lymphoma, distinguished by its "starry sky" histologic pattern. This pattern features tingible-body macrophages that contain apoptotic tumor cells, readily identifiable in the involved tissue. It also has a characteristic chromosomal translocation of the c-Myc gene from chromosome 8 to 14: t(8;14). This leads to a highly aggressive B-cell malignancy, commonly seen in children, regions endemic to malaria, and immunocompromised individuals [3]. There are distinct subtypes of Burkitt lymphoma, including endemic BL, predominantly affecting children in malaria-endemic regions, and sporadic BL, which occurs worldwide and is most common in children and young adults.

Although Hodgkin and non-Hodgkin lymphomas are typically distinct, rare instances exist where both types may coexist in a patient. These cases can present either as composite lymphoma, where both conditions are present in the same anatomical site, or as discordant lymphoma, where malignant processes occur in different sites either simultaneously or sequentially [4,5]. Composite lymphomas are exceptionally rare, with studies and clinical reviews consistently reporting their occurrence in only 1% to 4% of all lymphoma cases. Historical and recent literature provides robust data that support this range [6,7]. For instance, comprehensive reviews and case series analyses, which collectively examined hundreds of lymphoma cases, have found that composite lymphomas - those involving both Hodgkin and non-Hodgkin components - comprise a minor fraction of lymphoma diagnoses, solidifying their status as a rare clinical entity [8]. We report a rare case of composite lymphoma featuring both classical Hodgkin and Burkitt lymphoma within a single patient.

## Case presentation

A 42-year-old male with a one-year history of HIV and smoking, who is compliant with elvitegravir/cobicistat/emtricitabine/tenofovir highly active antiretroviral therapy (HAART), presented to the emergency department with progressive weight loss, low-grade fever, chills, night sweats, and lymphadenopathy, more in the neck, over four weeks. He stated his last viral load from seven months prior was reportedly undetectable, and his current CD4 count was 314 cells/mm³, though he had a CD4 count of 33 from a previous admission a year ago. Previously, he had sought care from his primary care provider for significant chest congestion and cough, for which he was prescribed a course of oral trimethoprim/sulfamethaxole (TMP/SMX) and amoxicillin/clavulanate. While his respiratory symptoms improved, his systemic symptoms persisted, prompting further evaluation at the hospital, considering his family history of diabetes and hypertension.

Upon examination, he was found to have stable vital signs except for hypotension (94/50 mmHg). Physical findings included significant bilateral anterior cervical lymphadenopathy, more pronounced on the left, and enlarged lymph nodes in both inguinal regions and the left axilla. Laboratory investigations showed pancytopenia and elevated alkaline phosphatase levels. Initial management included intravenous vancomycin, intravenous (IV) ondansetron for nausea, and continued oral TMP/SMX.

**Figure 1:**
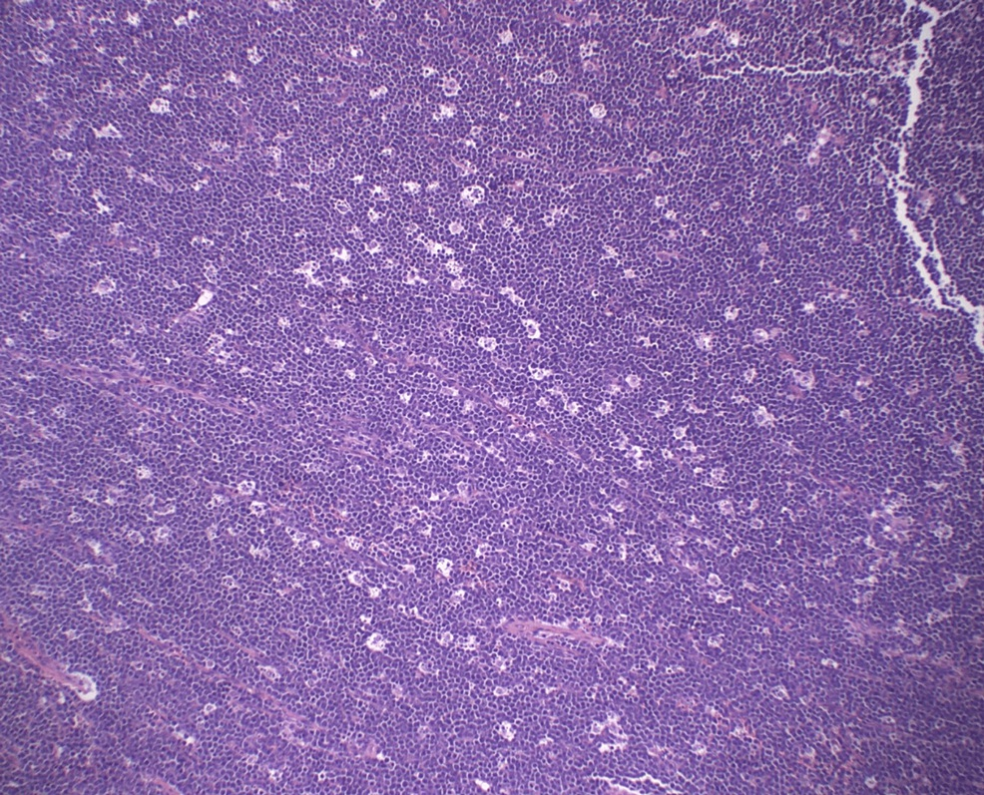
This patient's left inguinal excisional lymph node biopsy with H&E stain at 100x magnification shows a “starry sky” pattern characteristic of Burkitt lymphoma.

A left inguinal lymph node excisional biopsy was performed on the patient. Initial hematoxylin and eosin (H&E) staining at 100x magnification revealed a “starry sky” pattern characteristic of Burkitt lymphoma (Figure 1). The biopsy showed effacement of the normal lymph node architecture by a diffuse infiltrate of mostly intermediate-sized lymphocytes with round to slightly irregular nuclei, unevenly clumped to dispersed chromatin, distinct nucleoli, and squared-off cytoplasmic borders. High mitotic activity, apoptosis, and necrosis were observed, along with frequent tingible body macrophages, confirming the “starry sky” appearance typical of Burkitt lymphoma.

**Figure 2:**
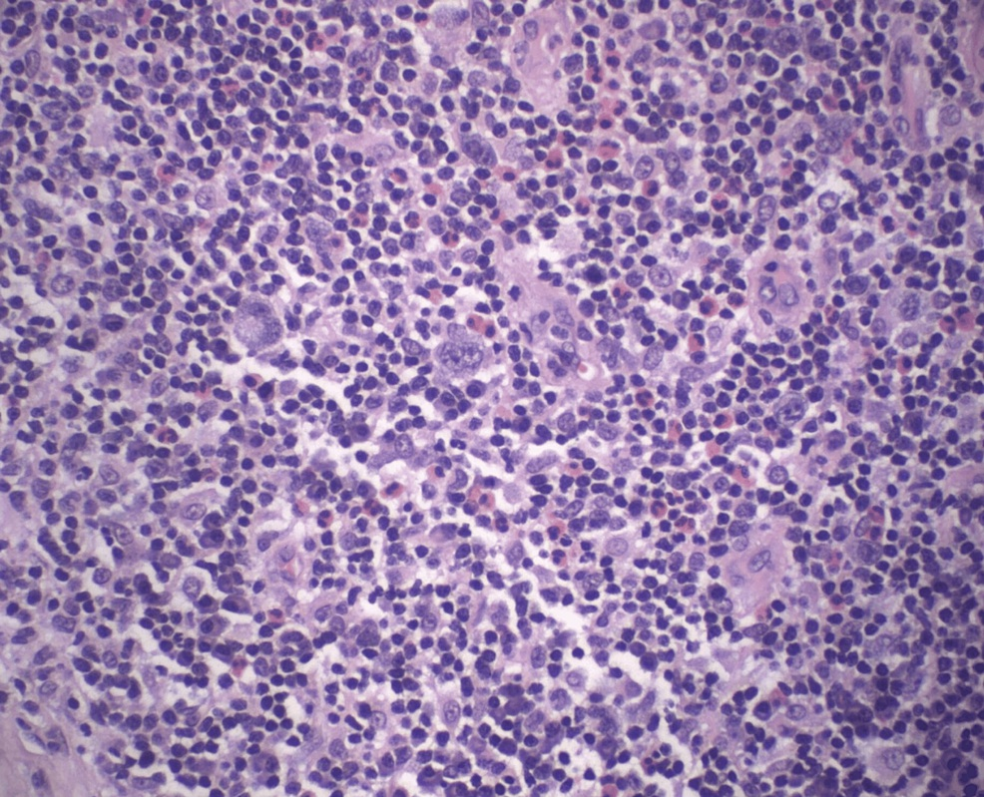
High-power magnification of this patient's left inguinal excisional lymph node biopsy with H&E stain at 400x magnification illustrates Reed-Sternberg and Hodgkin's cells accompanied by eosinophils, consistent with classical Hodgkin lymphoma.

A second infiltrative process was identified near one of the nodal poles. This process was characterized by a polymorphous cellular infiltrate, a slightly fibrous background, and scattered large cells consistent with Hodgkin/Reed-Sternberg (H/RS) cells (Figure 2).

 

**Figure 3:**
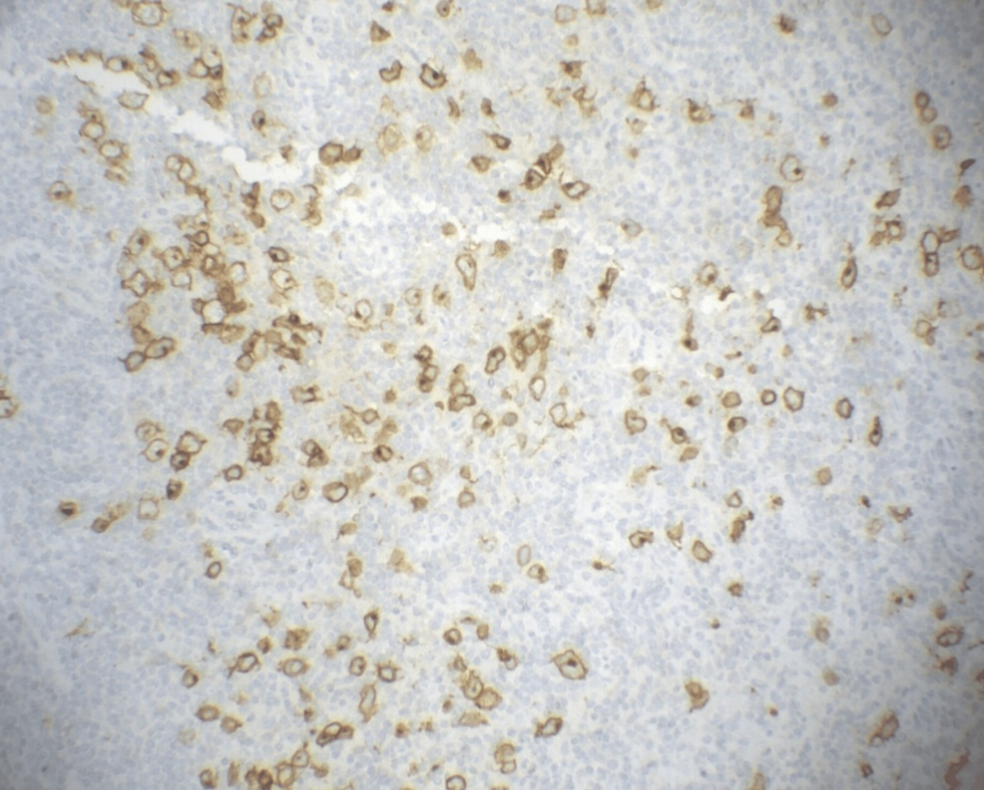
This patient's CD30 immunohistochemical staining of left inguinal lymph node tissue at 400x magnification highlighting Reed-Sternberg cells characteristic of classical Hodgkin lymphoma

Immunohistochemical (IHC) studies were conducted using markers CD3, CD10, CD20, BCL-2, Ki-67, and MUM1, and CD30 (Figure 3) and EBV (EBER). The predominant infiltrate was positive for CD20, CD10, and EBV, with a Ki-67 index near 100%, and negative for BCL-2 and MUM-1. The Hodgkin/Reed Sternberg-like cells in the second infiltrate were positive for CD30 and EBV.

Concurrent flow cytometry on the left inguinal lymph node revealed a cytoplasmic kappa light chain restricted monoclonal B-cell population coexpressing CD10. Fluorescent in situ hybridization (FISH) for the IGH/MYC translocation [t(8;14)(q24;q32)] was positive in 29% of interphase nuclei, supporting the diagnosis of Burkitt lymphoma. The combined morphological, immunophenotypic, and cytogenetic findings were consistent with involvement by both Burkitt lymphoma and classical Hodgkin lymphoma, both EBV-positive. A bone marrow biopsy showed hypercellular bone marrow with extensive (60%) involvement by EBV-positive malignancy showing morphologic and immunophenotypic features most consistent with classical Hodgkin lymphoma. It remained unclear to the physicians whether the culmination of these findings represented a "collision tumor" of two separately arising malignancies or a single EBV-driven process with bi-lineage differentiation, but a reduction of underlying immunosuppression was suggested as an important component of the treatment regimen.

Treatment was initiated with a dose-adjusted etoposide, prednisone, vincristine, cyclophosphamide, doxorubicin, and rituximab (DA-EPOCH-R) regimen to address the aggressive Burkitt lymphoma. Additionally, a low-dose rituximab infusion and intrathecal methotrexate were administered to target CD20-positive B-cell lymphomas and prevent central nervous system (CNS) involvement, respectively. The patient also received prophylactic measures against tumor lysis syndrome with oral allopurinol and intravenous (IV) fluids, and transfusions for anemia management. Following the start of systemic chemotherapy, vancomycin and ondansetron were discontinued as part of his treatment adjustment.

DA-EPOCH-R x 6 cycles from October 2014 to February 2015 achieved complete response for Burkitt lymphoma; follow-up bone marrow biopsy showed no residual disease but revealed new liver metastatic lesions found to be persistent Hodgkin's disease with confirmatory staining. This led to ICE x two cycles from March to April 2015, brentuximab x four cycles from April 28 to June 30, 2015, and gemcitabine 1000mg/m^2 and cisplatin 35mg/m^2 x four cycles from August to October 2015. Despite these treatments, Hodgkin's disease progression occurred with further interventions including nivolumab from December 19, 2015, to February 28, 2018, leading to additional complications such as sepsis, *Clostridium difficile* infections, and hepatic failure. A subsequent regimen of brentuximab vedotin 1.8 mg/kg plus bendamustine 90 mg/m^2 for six cycles from March to July 2018 achieved a complete response, followed by brentuximab maintenance (1.2 mg/kg) every 21 days from August 2018 to August 2019. The patient achieved sustained remission with no evidence of disease on subsequent scans, completing 18 cycles of brentuximab vedotin by August 2019. Ongoing clinical and lab monitoring continues, with CT scans showing remission of both lymphomas.

## Discussion

Due to its rarity, this case provides specific and valuable insights for future physicians. In the typical workup of a patient with suspected lymphoma, physicians often proceed under the assumption that a patient will only have one type of lymphoma. Traditionally, the presence of a composite lymphoma consisting of Hodgkin and non-Hodgkin lymphoma has been considered separate and unrelated malignancies. The challenge is compounded as the usual therapies for the typical young demographic prove potentially toxic in this age group [7]. Regardless of age, the presence of immunocompromised status adds another layer of caution for physicians in choosing the best course of action [7]. Subsequently, the decision-making process for the optimal initial and long-term treatment becomes challenging when there is no clear standard for such cases [8], as evident in our case.

In this instance, the physician prioritized treatment for the more aggressive of the two lymphomas, specifically the Burkitt lymphoma. The patient underwent combined treatment regimens, specifically DA-EPOCH-R, known for its efficacy in treating adult Burkitt lymphoma in patients with a history of HIV [9]. Additional regimens to consider for HIV-positive adults with Burkitt lymphoma could be rituximab, cyclophosphamide, vincristine, doxorubicin, high-dose methotrexate, ifosfamide, etoposide, and high-dose cytarabine (CODOX-M/IVAC-rituximab), a regimen that demonstrated a one-year progression-free survival of 69% in a recent AIDS Malignancy Consortium study involving HIV-positive patients [10]. However, in refractory cases of Burkitt, survival is less guaranteed, and stem-cell transplantation may be a potential consideration.

Even though the patient was treated with a myriad of chemotherapy agents shown to enhance the lifespan of patients with Hodgkin lymphoma, such as nivolumab and gemcitabine [11], the persistence of the disease was still a possibility, as it did occur in our case. In instances where a physician must address unforeseen cases of refractory Hodgkin lymphoma, the reality of HIV comorbidity and the risk of worsening this amidst the treatment of lymphoma has to be accounted for [12]. The subsequent regimen of brentuximab vedotin and bendamustine proved to be a safe and effective treatment for our patient. This combination has shown efficacy, particularly against refractory cases of Hodgkin lymphoma after initial therapy in HIV patients like ours [11-13]. Ultimately, the five-year journey presented by our case in treating composite lymphoma had some specific considerations. It required flexibility in combining standards of treatment for individual lymphomas and required by physicians to navigate and optimize care in the face of such unique manifestations of lymphoma. Currently, the patient continues to be monitored closely, and he is responding well to the latest therapeutic strategies with a stable condition, reflecting the careful clinical judgments made throughout his treatment journey.

## Conclusions

This case presents the diagnostic and therapeutic challenges of treating a 42-year-old male with concurrent Burkitt and classical Hodgkin lymphoma, underscoring the complexities physicians face with rare presentations. Key considerations include prioritizing treatment for the more aggressive lymphoma and adapting strategies to the patient's specific health context and lymphoma characteristics. This case emphasizes the importance of tailored treatment regimens, such as DA-EPOCH-R for Burkitt lymphoma and brentuximab vedotin with bendamustine for refractory Hodgkin lymphoma, highlighting the need for personalized and flexible approaches to manage complex lymphoma cases effectively. These insights are crucial for guiding future physicians in the nuanced management of unique and challenging lymphoma cases.
